# Dynamic Response of Fiber–Metal Laminates Sandwich Beams under Uniform Blast Loading

**DOI:** 10.3390/ma17184482

**Published:** 2024-09-12

**Authors:** Jianan Yang, Yafei Guo, Yafei Wu, Jianxun Zhang

**Affiliations:** 1 Basalt Fiber and Composite Key Laboratory of Sichuan Province, Dazhou 635000, China; jiananyang@stu.xjtu.edu.cn (J.Y.); w819270989@163.com (Y.W.); 2 State Key Laboratory for Strength and Vibration of Mechanical Structures, School of Aerospace Engineering, Xi’an Jiaotong University, Xi’an 710049, China; 3 Sichuan Sizhong Basalt Fiber Technology Research and Development Co., Ltd., Dazhou 635000, China; 4 State-Owned Luoyang Dancheng Wireless Power Plant, Luoyang 471000, China; yyzwzj86@163.com; 5 National Ceramic Industry Design Institute of China, Quanzhou 362500, China; 6 Key Laboratory of Mechanics on Disaster and Environment in Western China Attached to the Ministry of Education of China, Lanzhou University, Lanzhou 730000, China

**Keywords:** sandwich beam, FML, dynamic response, metal foam

## Abstract

In this work, theoretical and numerical studies of the dynamic response of a fiber–metal laminate (FML) sandwich beam under uniform blast loading are conducted. On the basis of a modified rigid-plastic material model, the analytical solutions for the maximum deflection and the structural response time of FML sandwich beams with metal foam core are obtained. Finite element analysis is carried out by using ABAQUS software, and the numerical simulations corroborate the analytical predictions effectively. The study further examines the impact of the metal volume fraction, the metal strength factor between the metal layer and the composite material layer, the foam strength factor of the metal foam core to the composite material layer, and the foam density factor on the structural response. Findings reveal that these parameters influence the dynamic response of fiber–metal laminate (FML) sandwich beams to varying degrees. The developed analytical model demonstrates its capability to accurately forecast the dynamic behavior of fiber–metal laminate (FML) sandwich beams under uniform blast loading. The theoretical model in this article is a simplified model and cannot consider details such as damage, debonding, and the influence of layer angles in experiments. It is necessary to establish a refined theoretical model that can consider the microstructure and failure of composite materials in the future.

## 1. Introduction

Fiber–metal laminates (FMLs) are recognized as up-to-date hybrid composite materials that merge the strengths of metals and fibers, offering superior strength and damage tolerance, making them ideal for critical applications in aerospace, automotive, defense, and infrastructure [1,2,3,4,5,6,7,8]. Sandwich structures, which are constructed with two rigid face sheets and a low-weight core, are favored for their high superior specific stiffness and strength, as well as their weight reduction and noise and thermal insulation capabilities, with core materials ranging from metal foams and lattice materials to woven and egg carton-like structures [9,10,11,12,13,14,15,16,17,18,19]. Thanks to their excellent performance and cost-effectiveness, these structures find extensive applications across various fields including construction, road transportation, rail transit, and aviation. Combined with the superior characteristics of fiber–metal laminates and sandwich panels (SPs), the new structure contemplated the integration of these two innovative structures to construct a new type of sandwich structure. Hence, a thorough exploration of FML sandwich beams under blast loading is required.

During the last several decades, a plethora of research work has been conducted on the dynamic response (DR) of sandwich structures under explosive loading. For example, Fleck et al. [20] established an analytical model for the DR of clamped sandwich beams with metal foam cores under impulsive loading, and obtained the bounds of large deflections of sandwich beams. Imbalzano et al. [21] designed a novel theoretical model for assessing the capability of SPs with auxiliary cellular cores and metal face sheets under explosive blast, and their findings suggested that auxiliary SP could absorb more impact energy compared to single metal plates. Zhou et al. [22] utilized a combination of theoretical analysis, experimental studies, and finite element simulations to explore the DR of the clamped square SPs with gradient foam cores under blast loading (BL) and revealed that SPs featuring a negatively graded core exhibit superior energy absorption, in comparison to those with a positively graded core. Zhu et al. [23] explored the deformation/damage mechanisms and the DR of square metal SPs under explosive loading through experimental and numerical analyses. Li et al. [24] examined the DR of stepwise gradient aluminum cellular core sandwich structures under explosive loading using a combination of a spherical pendulum experimental setup and finite element simulation, and the results indicate that SPs with a core arrangement decreasing relative density display superior protective capability and energy dissipation efficiency. Karagiozova et al. [25] utilized experiments and digital simulations to analyze the mechanical properties of steel-faced SP with polystyrene cores under explosive loading, and found that SPs with polystyrene cores were more efficient in energy absorption, particularly with thicker cores. Shen et al. [26] experimentally explored the DR of curved composite panels with distinct curvature radii and core/face sheet configurations under BL, and found that the initial curvature of the curved SP could alter their deformation/collapse modes. Liu et al. [27] experimentally explored the performance of steel SP with an aluminum foam core under BL, discovering that SPs could reduce the peak load by about 60% compared to steel plates. Arora et al. [28] analyzed the dynamic response of glass fiber-reinforced polymer composite SPs under multiple and single blast impacts by means of experiments and finite element calculations, and discovered that the SP exhibits significant protective capability. Xue et al. [29] numerically explored the DR of SP under BL, and found that metal SPs with a sufficiently strong core could withstand significantly higher explosive pressures compared to solid plates of identical material and mass. Varghese et al. [30] conducted numerical simulations to study the dynamic response of SPs with triangularly woven and pyramidal lattice cores under blast loading, and found that pyramidal lattice core SPs exhibit enhanced protective capability under both normal and consecutive BL compared to their triangularly woven counterparts. Soutis et al. [31] established a numerical model using the ConWep algorithm and multi-material Arbitrary Lagrangian Eulerian (MMALE) method to investigate the dynamic response of fully clamped GLARE panels subjected to blast loads, and validated the effectiveness of the model through comparison with experimental data, indicating that GLARE panels exhibit superior protective capability compared to conventional aluminum panels. Langdon et al. [32] used the ballistic pendulum to conduct blast tests of GLARE panels, and they found that the deflection under specific non-dimensional impacts was approximately 11% less than that of traditional metal plates, indicating its potential as a protective material.

As contemporary manufacturing techniques keep progressing and being refined, FML sandwich structures have become a reality, and some scholars have investigated the structural characteristics of FML sandwich structures. Zhang et al. [33] explored the plastic behavior of FML sandwich beams with metal foam cores under transverse loading through analytical and numerical simulation methods. Yuan and Zhang [34] studied the DR of slender multilayer FML foam-cored sandwich beams under low-speed impacts through theoretical analysis and numerical simulation, showing high consistency between theoretical and numerical results. Liu et al. [35] examined the effects of foam core thickness and FML skin on the impact response of FML SPs through drop-weight impact tests and numerical calculations, and the findings indicated that increasing foam core and FML skin thickness could enhance the energy absorption of SP. Liu et al. [36] explored the high-speed impact of FML SPs with an aluminum foam core using gas gun tests, and the results demonstrate that appropriately increasing the thickness of FML face sheets can effectively enhance the energy absorption capacity. Ma et al. [37] carried out experimental studies on the DR of FML-faced graded aluminum honeycomb SPs subjected to explosive impacts, demonstrating that FML face sheets significantly bolster protective capability. Kong et al. [38] established an analytical model for the dynamic response and failure behavior of thermoplastic fiber–metal laminates (TFMLs) under confined blast loads, and used digital image correlation (DIC) technology and X-ray computed tomography (CT) imaging to analyze the deformation process and damage mechanisms of the laminates. Baştürket et al. [39] anticipated the DR of an aluminum foam-cored FML sandwich beam under explosive loading. Reyes [40] explored the DR of SP composed of thermoplastic FML face sheets and aluminum foam cores under low-speed impacts experimentally, indicating substantial energy absorption of the sandwich configuration.

**Figure 1:**
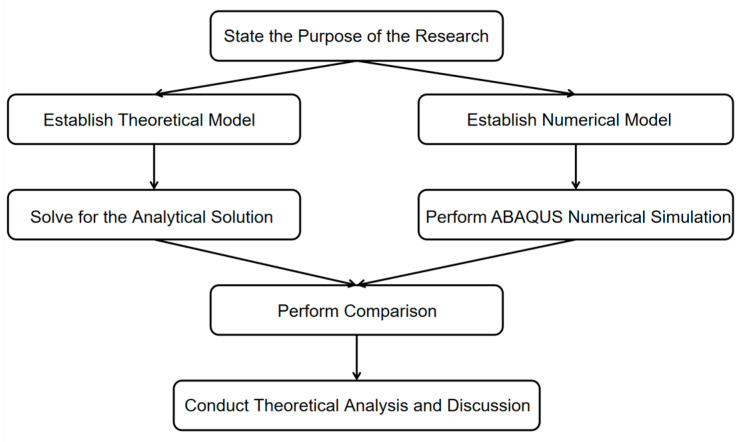
Flowchart of theoretical and numerical research steps.

To better predict the DR of FML sandwich beams with metal foam cores under uniform BL, such as maximum deflection and structural response time in relation to certain mechanical properties, this work proposes a theoretical model of the DR of FML sandwich beams under BL both theoretically and numerically. The problem formulation is described in Section 2. Analytical solutions for the DR of the FML sandwich beam under uniform BL are obtained in Section 3. Finite element analysis is presented in Section 4. Analytical predictions are compared to their numerical counterparts. The effects of the metal volume fraction, the metal strength factor, the foam strength factor, and the foam density factor on structural response are discussed in Section 5. The comprehensive conclusions are outlined in Section 6. Figure 1 shows a flowchart of theoretical and numerical research steps.

## 2. Problem Formulation

**Figure 2:**
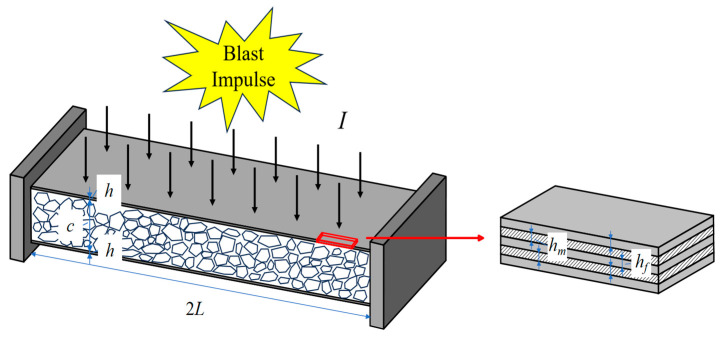
Schematic of a fully clamped FML sandwich beam with metal foam cores under uniform BL.

Consider a completely fixed FML sandwich beam with metal foam cores under uniform BL, as shown in Figure 2, with a length of 2L. The sandwich beam contains three parts, i.e., the upper and lower face sheets, and the intervening metal foam core. The face sheets are constructed from FML and possess a total thickness, denoted by h. The FMLs consist of n metal layers with a thickness of $h_{m}$ and (n−1) composite material layers with a thickness of $h_{f}$, so that

(1) $$h=nh_{m}+\left(n-1\right)h_{f}$$

The core of the sandwich beam is a metal foam layer with thickness c.

The metal layer is characterized by a density of $\rho_{m}$, yield strength of $\sigma_{m}$, and Young’s modulus of $E_{m}$. The composite material layer possesses the density of $\rho_{f}$, yield strength of $\sigma_{f},$ and Young’s modulus of $E_{f}$. For the isotropic metal foam core, the density is $\rho_{c}$, the yield strength is $\sigma_{c}$, and the Young’s modulus is $E_{c}$.

To enhance the precision in characterizing the strength ratio between the metal and composite material layers, a metal strength factor, referred to as q, is introduced to quantify this relationship.

(2) $$q=\frac{\sigma_{m}}{\sigma_{f}}$$

For 0<q<1, the metal layer’s strength in the sandwich beam is inferior to the composite material layer’s. Conversely, a strength predominance of the metal layer is reflected when q>1, relative to the composite material layer. q=1 reflects an identical strength between two layers.

Moreover, to evaluate the density relationship between the metal and composite material layer, a metal density factor is defined as $\bar{m}$

(3) $$\bar{m}=\frac{\rho_{m}}{\rho_{f}}$$

## 3. Analytical Solutions

In the event that plastic deformation takes precedence in the plate’s response, theoretical solutions based on rigid-plastic behavior can be utilized to predict the DR of FML [41]. In this instance, the process is extended to anticipate the DR of FML sandwich beams under uniform BL.

Zhang et al. [42] explored a theoretical analytical model for the DR of sandwich beams with a metal foam core under BL, according to the plastic-string model. In the interest of completeness, the structural responses of sandwich beams to explosive loading are concisely discussed. The metal sandwich beams include identical top and bottom metal face sheets of thickness *h*, with a metal foam core of thickness c in between. The mass of the beam for each unit length is $G_{b}$, and the beam’s length is 2L. The impulse I is imposed on the upper face sheet of the sandwich beam. The face sheets adhere to the rigid-perfectly plastic constitutive model, with a yield stress designated as $\sigma_{fa}$. Conversely, the metal foam core is hypothesized to be composed of a rigid-perfectly plastic locking material, featuring a yield strength denoted as $\sigma_{c}$ and undergoing a transition to densification at a characteristic strain level $\varepsilon_{D}$.

In the case of significant deflection for the sandwich beam, the impact of the bending moment can be entirely disregarded, with the DR being solely governed by the axial (membrane) force. The governing equations for the sandwich beam system can be formulated as follows [42]:

(4) $$N_{p}^{\prime }W_{0}=-\frac{d}{dt}\int_{0}^{L}(2\rho_{fa}bh+\rho_{c}bc)\dot{Wzdz}$$

where $N_{p}$ represents the longitudinal plastic membrane force, $\dot{W}$ indicates the velocity of the sandwich beam, $W_{0}$ denotes the deflection at the midpoint of the sandwich beam, $\rho_{fa}$ is the weighted density of the FML, and b  refers to the width of the sandwich beam.

For the sake of simplicity and to facilitate the ensuing analysis, the following dimensionless variables are hereby introduced:

$$\bar{L}=\frac{L}{c},\;\bar{h}=\frac{h}{c},\;\bar{\rho_{c}}=\frac{\rho_{c}}{\rho_{m}},\;\bar{\sigma_{c}}=\frac{\sigma_{c}}{\sigma_{m}},\;\bar{W_{0}}=\frac{W_{0}}{2h+c},\;\bar{T_{f}}=\frac{T_{f}}{L\sqrt{\frac{\rho_{m}}{\sigma_{m}}}},\;\bar{I}=\frac{I}{(2\rho_{m}h+\rho_{c}c)\sqrt{\frac{\sigma_{m}}{\rho_{m}}}}.$$

Using the linear velocity field, the maximum deflection and structural response time of metal sandwich beams under uniform BL are given by [42]

(5) $$\bar{W_{0}}=\frac{\bar{I}\bar{L}\;\beta }{\sqrt{3}(1+2\bar{h})}$$

and

(6) $$\bar{T_{f}}=\frac{\pi }{2\sqrt{3}}\beta$$

where

$$\beta =\sqrt{\frac{\bar{\rho_{c}}+2\bar{h}}{\bar{\sigma_{c}}+2\bar{h}}},\;\bar{T_{f}}=\frac{T_{f}}{T_{c}},\;T_{c}=L\sqrt{\frac{\rho_{m}}{\sigma_{m}}},\;\bar{I}=\frac{I}{I_{c}},\;I_{c}=(2\rho_{m}h+\rho_{c})\sqrt{\frac{\sigma_{m}}{\rho_{m}}}.$$

First, the metal volume fraction (MVF) is defined as follows [41]:

(7) $$f=\frac{nh_{m}}{h}\times 100\%$$

Considering the varying yield strengths and thicknesses of the FML components, the weighted strength, denoted as $\sigma_{fa}$, is given by

(8) $$\sigma_{fa}=f\sigma_{m}+(1-f)\sigma_{f}$$

Furthermore, taking into account the different densities and thicknesses of the elements of the FML, the weighted density, denoted as $\rho_{fa}$, is given by [41]

(9) $$\rho_{fa}=f\rho_{m}+(1-f)\rho_{f}$$

Substituting Equations (8) and (9) into Equations (5) and (6), a theoretical solution for the maximum deflection of the FML sandwich beam under uniform BL is obtained.

(10) $$\bar{W_{0}}=\frac{\bar{L}}{1+2\bar{h}}\sqrt{\frac{2\bar{h}+\frac{\bar{\rho }}{f\left(\bar{m}-1\right)+1}}{3(2\bar{h}+\frac{\bar{\sigma }}{f\left(q-1\right)+1})}}\cdot \bar{I}$$

where

$$\bar{\rho }=\frac{\rho_{c}}{\rho_{f}},\;\bar{\sigma }=\frac{\sigma_{c}}{\sigma_{f}},\;\bar{I}=\frac{I}{I_{c}^{\prime }},\;I_{c}^{\prime }=(2\rho_{fa}h+\rho_{c}c)\sqrt{\frac{\sigma_{fa}}{\rho_{fa}}}.$$

The structural response time $\bar{{\;\;T}_{f}}$ of the FML sandwich beam under uniform BL can be expressed as

(11) $$\bar{T_{f}}=\frac{\pi }{2}\sqrt{\frac{2\bar{h}+\frac{\bar{\rho }}{f\left(\bar{m}-1\right)+1}}{3(2\bar{h}+\frac{\bar{\sigma }}{f\left(q-1\right)+1})}}$$

where

$$\bar{T_{f}}=\frac{T_{f}}{T_{c}^{\prime }},\;T_{c}^{\prime }=L\sqrt{\frac{\rho_{fa}}{\sigma_{fa}}}.$$

## 4. Finite Element Analysis

In this section, the numerical simulations of the DR of FML sandwich beams under uniform BL are performed utilizing the ABAQUS/Explicit software version 2023 [43]. Both the FML panels and the metal foam core are modeled using lower-order integrated 8-node brick elements (C3D8R). The sandwich beam is appropriately refined with meshing to ensure computational accuracy. Furthermore, the mesh sensitivity of the model is examined, and the simulation results show that further mesh refinement has no substantial effect on the numerical results.

The sandwich beam has a half-span length of L  = 200 mm. The thickness of the metal foam core material is c  = 6 mm, i.e., $\;\bar{c}=c/L=0.03$. The thicknesses of the metal and composite material layers are $h_{m}\;$ = 0.5 mm and $h_{f}\;$ = 0.2 mm. With n=3 layers of metal and two layers of composite material, the total thickness of the FML layers is h  = 1.9 mm. Two cases are considered, i.e., b  = 50 mm and b  = 100 mm.

Based on empirical evidence [44], the woven glass composite layers within the FML are constituted by a quasi-isotropic glass fiber fabric, which is evenly distributed in the 0, +45°, 90°, and −45° directions, with the interstices of the fibers filled with vinyl ester resin. The physical properties of this composite material layer are as follows: density of $\rho_{f}$ = 1700 kg/m^3^, yield strength of $\sigma_{f}\;$ = 220 MPa, Young’s modulus of $E_{f}\;$ = 10 GPa, and elastic Poisson’s ratio of $v_{ef}=0.3$. For the fiber-reinforced metal laminate (FML) in the composite sandwich structure, the material is presumed to be linearly elastic during tensile loading [40]. The metal layers of the FML have the following mechanical properties: yield strength of $\sigma_{m}\;$ = 460 MPa, Young’s modulus of $E_{m}\;$ = 70 GPa, elastic Poisson’s ratio of $v_{em}=0.3$, and density of $\rho_{m}\;$ = 2800 kg/m^3^. The behavior of the metal layers is governed by a linear hardening principle with a modulus of $E_{mt}=0.02E_{m}$ in the tangent direction. It is further supposed that the metal layers possess sufficient ductility to avoid fracture during the deformation process.

The depiction of plastic deformation and crushing attributes of metal foam in the ABAQUS software is achieved through the application of the Deshpande–Fleck model [45]. This model accounts for alterations in the yield surface geometry, which are a consequence of distinct hardening phenomena along the hydrostatic and deviatoric stress paths. The yield criterion of the foam is given by

(12) $$\phi =\hat{\sigma }-\sigma_{c}=0$$

where

(13) $${\hat{\sigma }}^{2}\equiv \frac{1}{1+{(\alpha /3)}^{2}}(\sigma_{e}^{2}+\alpha^{2}\sigma_{s}^{2})$$

The von Mises effective stress $\sigma_{e}$ is defined by the expression $\sigma_{e}=\sqrt{3s_{ij}s_{ij}/2}$, where $s_{ij}$ denotes the deviatoric stress components. This measure encapsulates the intensity of shear stress within a material. Concurrently, the mean stress is determined from the summation of these deviatoric components. Furthermore, the shape factor of the yield surface $\sigma_{s}$ is delineated as $\sigma_{s}=\sigma_{kk}/3$, with $\sigma_{kk}$ representing the hydrostatic stress. Employing the associated plastic flow rule, the plastic Poisson’s ratio $v_{p}$ is ascertained as follows:

(14) $$v_{p}=-\frac{{\dot{\varepsilon }}_{22}^{p}}{{\dot{\varepsilon }}_{11}^{p}}=\frac{1/2-{\left(\alpha /3\right)}^{2}}{1+{\left(\alpha /3\right)}^{2}}$$

The isotropic metal foam core has the following mechanical properties: yield strength of $\sigma_{c\;}$ = 10 MPa, Young’s modulus of $E_{c}\;$ = 10 GPa, elastic Poisson’s ratio of $v_{ec}=0.3$, plastic Poisson’s ratio of $v_{p}=0$, and density of $\rho_{c}\;$ = 405 kg/m^3^. The metal foam exhibits a stress plateau of $\sigma_{c}$ in its stress–strain curve, up to a densification strain of $\varepsilon_{D}=0.5$. Upon reaching the point beyond densification, the metal foam complies with a linear hardening pattern characterized by a tangent modulus of $E_{ct}=0.02E_{m}$.

## 5. Results and Discussion

**Figure 3:**
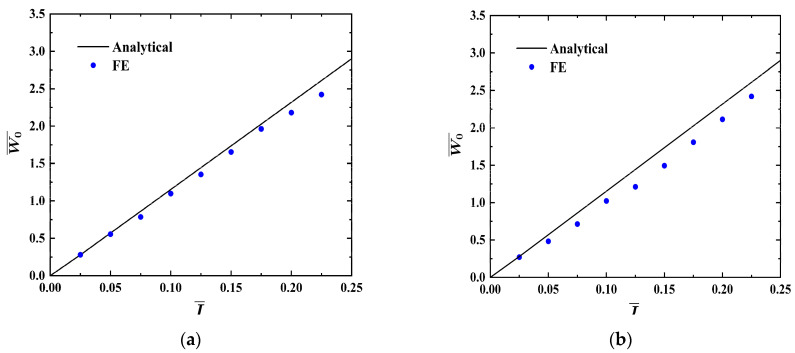
Analytical and numerical results of the dimensionless maximum deflection $\bar{W_{0}}$ vs. impulse $\bar{I}\;$ curves of FML sandwich beams subjected to uniform BL. (**a**) *L* = 200 mm, *b* = 50 mm, (**b**) *L* = 200 mm, b = 100 mm.

Figure 3a,b show analytical and numerical results of the dimensionless maximum deflection $\;\bar{W_{0}}$ vs. impulse $\bar{I}$ curves of the FML sandwich beams under uniform BL. In Figure 3a, L  = 200 mm and b = 50 mm; in Figure 3b, L = 200 mm and b = 100 mm. It can be seen that the analytical predictions align well with numerical ones, with the analytical findings being slightly larger than the numerical ones. The gap between analytical and numerical results could be attributed to the disregard for material elasticity and strain hardening in the analytical calculations.

**Figure 4:**
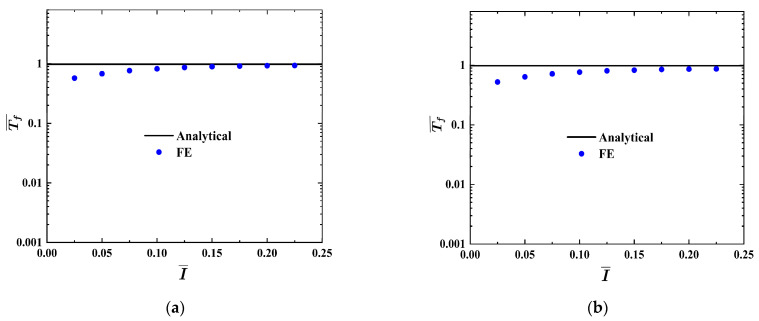
Analytical and numerical results of the dimensionless structural response time $\bar{T_{f}}\;$ vs. impulse $\bar{I}\;$ curves of FML sandwich beams subjected to uniform BL. (**a**) L  = 200 mm, b  = 50 mm, (**b**) L  = 200 mm, b  = 100 mm.

The analysis and numerical results of the structural response time $\bar{T_{f}}$ vs. impulse $\bar{I}$ curves of the FML sandwich beams under uniform BL are shown in Figure 4a,b. It can be seen that the numerical response time $\bar{T_{f}}$ of the FML sandwich beam is a little lower than the analytical one in the low impulse stages. When the impulse $\bar{I}$ increases, the structural response time $\bar{T_{f}}\;$ tends to stabilize, and there is a close correspondence between analytical and numerical results.

**Figure 5:**
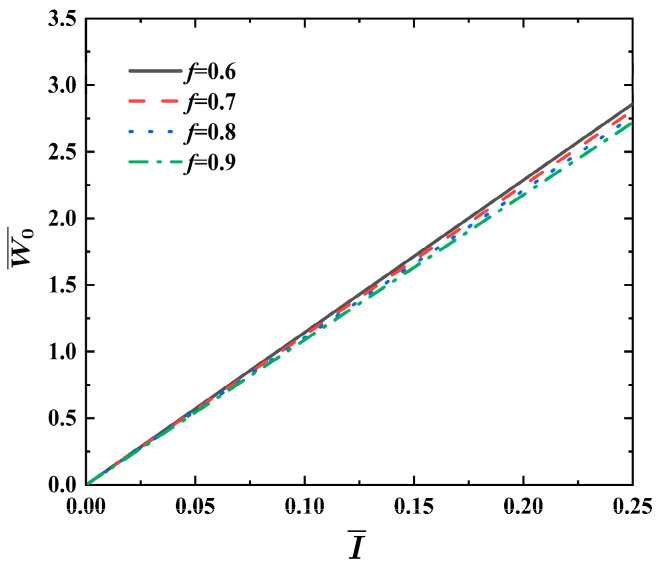
Effects of MVF *f* on the dimensionless maximum deflection $\bar{W_{0}}$ vs. impulse $\bar{I}$ curves of FML sandwich beams subjected to uniform BL.

Figure 5 illustrates the effect of the MVF *f* on the dimensionless maximum deflection $\;\bar{{\;W}_{0}}$ vs. impulse curves of FML sandwich beams under uniform BL, where $\bar{\sigma }=0.04$, q=2, $\bar{\rho }=0.2$, $\bar{h}=0.4$, and n=3. As MVF *f* increases, the dimensionless maximum deflection $\bar{W_{0}}$ of the FML sandwich beam decreases for the given impulse $\bar{I}$. It is found that a rise in MVF *f* contributes minimally to the ability of the sandwich beam to withstand impacts.

**Figure 6:**
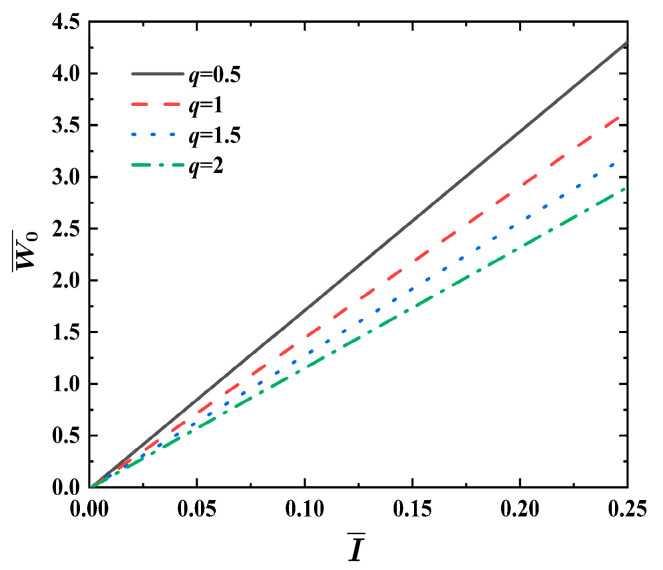
Effects of metal strength factor q on the dimensionless maximum deflection $\bar{W_{0}}$ vs. impulse $\bar{I}\;$ curves of FML sandwich beams subjected to uniform BL.

Figure 6 illustrates the effects of the metal strength factor q on the dimensionless maximum deflection $\bar{W_{0}}$ relative to impulse $\bar{I}$ for FML sandwich beams under uniform BL, where $\bar{\sigma }=0.04$, f=0.6, $\bar{\rho }=0.2$, $\bar{h}=0.4$, and n=3. For the given impulse $\bar{I}$, the dimensionless maximum deflection $\bar{W_{0}}$ of the FML sandwich beam lessens with the increase in the metal strength ratio q. It can be seen that an increment in the ratio q is almost linearly related to the decrease in the dimensionless maximum deflection $\bar{W_{0}}\;$ of the FML sandwich beam. The increase in the metal strength ratio q has a significant effect on enhancing the dynamic response of the FML sandwich beam.

**Figure 7:**
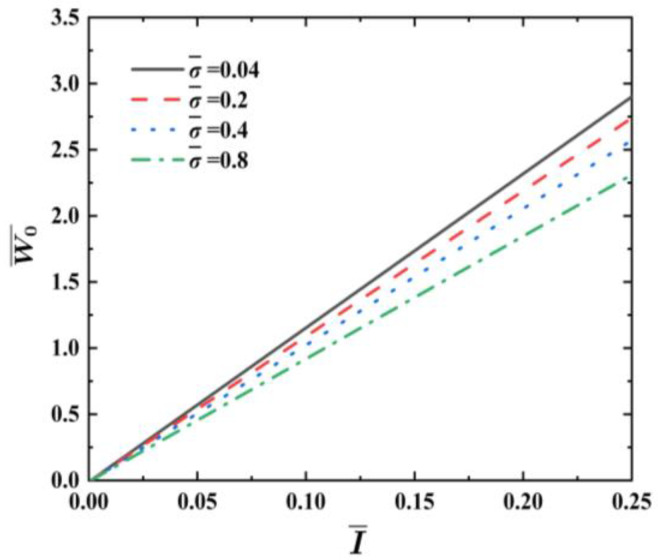
Effects of foam strength factor $\bar{\sigma }$ on the dimensionless maximum deflection $\bar{W_{0}}$ vs. impulse $\bar{I}$ curves of FML sandwich beams subjected to uniform BL.

Figure 7 illustrates the effects of the foam strength factor $\bar{\sigma }$ on the dimensionless maximum deflection $\bar{W_{0}}$ vs. impulse $\bar{I}$ curves of FML sandwich beams under uniform BL, where q=2, f=0.6, $\bar{\rho }=0.2$, $\bar{h}=0.4$, and n=3. It is apparent that the dimensionless maximum deflection $\bar{W_{0}}$ of the FML sandwich beams lessens in response to an increase in $\bar{\sigma }$ for the designated impulse $\bar{I}$. The increment in the foam strength factor $\bar{\sigma }$ is almost linearly related to the decrease in the dimensionless maximum deflection $\bar{W_{0}}$ of the FML sandwich beam.

**Figure 8:**
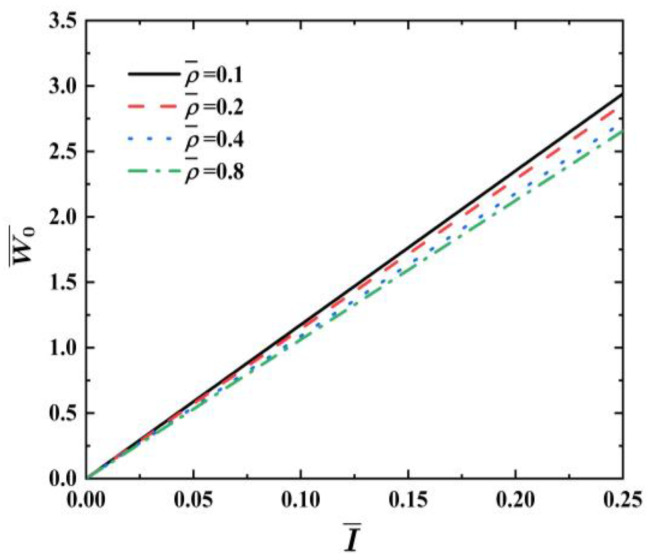
Effects of foam density factor $\bar{\rho }$ on dimensionless maximum deflection $\bar{W_{0}}$ vs. impulse curves of FML sandwich beams subjected to uniform BL.

Figure 8 illustrates the effects of the foam density factor $\bar{\rho }$ on the dimensionless maximum deflection $\bar{W_{0}}$ vs. impulse $\bar{I}$ curves of FML sandwich beams under uniform BL, where $\bar{\sigma }=0.04$, q=2, f=0.6, $\bar{h}=0.4$, and n=3. It is evident that an increase in the foam density $\bar{\rho }$ results in a decrease in the dimensionless maximum deflection $\bar{{\;W}_{0}}$ for FML sandwich beams under a defined impulse. The foam density $\bar{\rho }$, when increased, results in a minimal improvement in the dynamic response of the FML sandwich beam.

**Figure 9:**
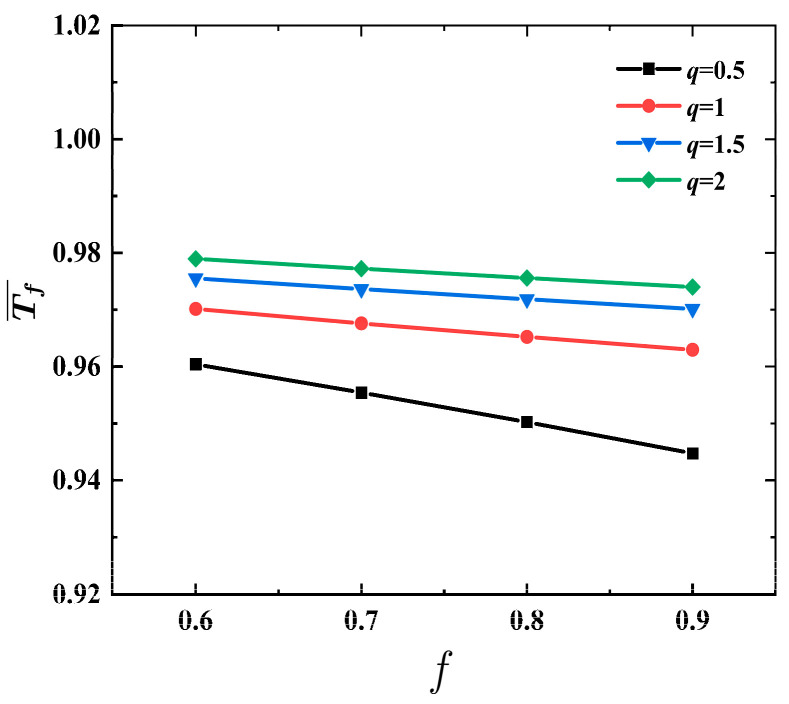
Effects of metal strength factor q on structural response time $\bar{T_{f}}$ vs. MVF *f* of the FML sandwich beam subjected to uniform BL.

Figure 9 shows the effects of metal strength factor q on structural response time $\bar{T_{f}}$ vs. MVF *f* of FML sandwich beam under uniform BL, where $\bar{\sigma }=0.04$, $\bar{\rho }=0.2$, $\bar{h}=0.4$, and n=3. When MVF *f* increases, the structural response time $\;\;\bar{T_{f}}$ of the FML sandwich beam almost decreases linearly. For the same given MVF *f*, the structural response time $\bar{{\;\;T}_{f}}\;$ of FML sandwich beams is seen to escalate as the metal strength ratio q increases. Moreover, the difference between q=0.5 and q=1 is evidently bigger than in other cases.

**Figure 10:**
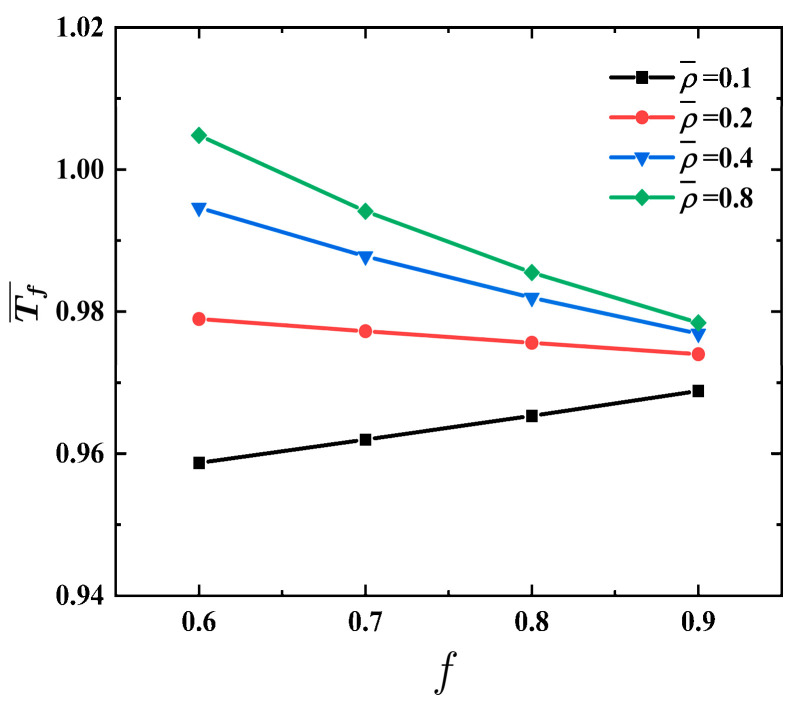
Effects of foam density factor $\bar{\rho }\;$ on structural response time $\bar{T_{f}}$ vs. MVF *f* of the FML sandwich beam subjected to uniform BL.

Figure 10 shows the effects of the foam density factor $\bar{\rho }\;$ on structural response time $\bar{T_{f}}$ vs. MVF *f* of the FML sandwich beam under uniform BL, where $\bar{\sigma }=0.04$, q=2, $\bar{h}=0.4$, and n=3. When the foam density $\bar{\rho }$ is set at 0.2, the structural response time $\bar{T_{f}}$ remains relatively stable. Furthermore, for lower foam density $\bar{\rho }$, there is a little increase in the structural response time $\bar{T_{f}}$ as MVF *f* increases. Conversely, for foam density $\bar{\rho }$, the rise in structural response time $\bar{T_{f}}$ is considerably less pronounced as MVF *f* increases.

## 6. Concluding Remarks

In the current study, we conduct an analytical and numerical exploration of the DR of FML sandwich beams under uniform BL. Utilizing an enhanced rigid-plastic material approximation, a simple formula for the maximum deflection and time response of foam core FML sandwich beams is obtained. The predictions derived analytically show a satisfactory correspondence with numerical data. It is evidenced that the maximum deflection of the FML sandwich beam decreases correspondingly with the increase of MVF *f*, metal strength factor q, foam strength factor, and foam density factor. Furthermore, the research suggests that the structural response time does not change significantly with the alteration of MVF and metal strength factor. The current theoretical model can effectively predict the DR of FML sandwich beams under uniform BL. Moreover, this work enables a theoretical analysis of the relationship between maximum deflection, structural response time, and certain mechanical properties, providing a qualitative understanding of the influencing trends. The present theoretical model is a simplified model and cannot consider details such as damage, debonding, and the influence of layer angles in experiments. It is necessary to establish a refined theoretical model that can consider the microstructure and failure of composite materials in the future.

## Data Availability

The authors confirm that all data for this study are included in the paper.
